# Editorial: Early indicators of cognitive decline, Alzheimer's disease, and related dementias captured by neurophysiological tools

**DOI:** 10.3389/fpsyg.2024.1393724

**Published:** 2024-04-09

**Authors:** Alexandra Wolf, Karine Ravienna, Elena Salobrar-Garcia

**Affiliations:** ^1^Cognitive Behavioral Assistive Technology (CBAT), Goal-Oriented Technology Group, RIKEN Center for Advanced Intelligence Project (AIP), Tokyo, Japan; ^2^Department of Neuropsychiatry, Graduate School of Medical Sciences, Kyushu University, Fukuoka, Japan; ^3^Vanaya NeuroLab Brain and Behavior Research Center, Jakarta, Indonesia; ^4^Instituto de Investigaciones Oftalmológicas Ramón Castroviejo, Grupo UCM 920105, IdISSC, Universidad Complutense de Madrid, Madrid, Spain; ^5^Facultad de Óptica y Optometría, Departamento de Inmunología, Oftalmología y ORL, Universidad Complutense de Madrid, Madrid, Spain

**Keywords:** biomarker, cognitive scoring, dementia, EEG, eye-tracking, mild cognitive impairment (MCI), neurophysiology, optical coherence tomography angiography (OCTA)

Major neurocognitive disorders represent a significant global challenge, impacting a substantial number of individuals and placing emotional and financial strain on caregivers (Werner, 2012; World Health Organization, 2017; Wright and O'Connor, 2018). In response to the imperative requirement for early detection and intervention, the editors of this Research Topic aimed to enhance comprehension of brain integrity, concentrating mainly on biomarker investigations targeting timely identification of the mild cognitive impairment (MCI) stage. As an accelerated cue, traditional diagnostic trajectories for dementia are often initiated with standardized cognitive assessments like the Mini-Mental State Examination and Montreal Cognitive Assessment (Wolf and Ueda, 2021; López-Cuenca et al., 2022; Whelan et al., 2022); yet their limited sensitivity to subtle cognitive impairments has become increasingly pronounced. Furthermore, the influence of pandemic-related lockdowns and lockdown-like measures during conflicts on dementia diagnosis rates cannot be overlooked (Bick and Nelson, 2016; Brown et al., 2020; Tani et al., 2020; Ismail et al., 2021; Corney et al., 2022; Górski et al., 2022). The global disruptions trigger a significant decline in individuals seeking treatment for dementia-related symptoms, resulting in dementia diagnoses falling below anticipated levels (Axenhus et al., 2022; Hazan et al., 2023). This highlights the urgent necessity for innovative yet cost-effective early-stage intervention strategies to adeptly confront the swiftly evolving global challenge (Irazoki et al., 2020; Tokunaga et al., 2021; Braun et al., 2024).

In this context, we recognize the urgency for practical strategies that will bridge subjective evaluations with objective physiological metrics. By presenting insights from 10 original research projects and three reviews, all focused on early indicators of cognitive decline, Alzheimer's disease (AD), and related dementias, our Research Topic holds the potential for significant individual and societal benefits. Synthesizing findings from various specializations (e.g., visual processing, language impairments) and techniques (EEG, eye-tracking, or optical coherence tomography angiography), this work addresses the multifaceted challenges associated with a dementia diagnosis and paves the way for more effective early intervention initiatives.

The study conducted by Ma et al. employed optical coherence tomography angiography to examine retinal vascular changes in AD and MCI. Significantly, their findings suggested that these alterations in retinal microvasculature hold potential as promising biomarkers for AD and MCI. Plaza-Rosales et al., on the other hand, contribute to the nuanced understanding of the visual narrative by examining visual-spatial processing in the initial stages of AD. The investigators employed a spatial navigation task, incorporating comprehensive behavior recordings, EEG, and eye-tracking. This research portends clinical promise for early diagnostic applications, thereby holding considerable importance in enhancing the quality of life for affected individuals and easing the associated healthcare costs. To zoom into the visual integration domain, Elvira-Hurtado et al. enhance the understanding of AD by investigating its continuum with visual implications, revealing the intricate interplay between visual factors and cognitive progression. Compared to a control group, the study assessed visual function differences at different AD stages, i.e., family history group (FH+), mild cognitive impairment (MCI), mild AD, and moderate AD. The results showed a significant decrease in visual acuity, contrast sensitivity, and visual integration scores in MCI, mild AD, and moderate AD groups. Notably, the research group underlined the utility of visual psychophysical tests alongside neuropsychological assessments as valuable tools for early AD diagnosis. Following that, posing that implementing automated electronic reports tailored to clinical needs will ensure swift responsiveness to patient requirements, Huang, Zhang et al. introduce the vestibular cognition assessment system (VCAS). This practical advancement provides a framework for improving visuospatial cognition in individuals with vestibular impairment, portraying VCAS as a potent instrument for comprehending the intricate interplay between spatial memory, navigation, and cognitive proficiency.

Language is intricately linked to brain function, making cognitive impairment a potential cause of language disorders (Baldo et al., 2015; Dronkers et al., 2017; Abe and Otake-Matsuura, 2021). Therefore, shifting readers' attention to language and memory, Kong et al. explore the role of spoken discourse in episodic autobiographical and verbal short-term memory. This linguistic investigation enriches understanding of cognitive functions beyond the visual domain, integrating language into the spectrum of cognitive research. The study highlights the intricate relationship between coherence in personal narrative and episodic autobiographical memory, suggesting potential interventions through conversation. Additionally, the research team identified indices like global coherence, informativeness, and empty speech as potential markers of memory functions in individuals with cognitive impairments. In light of other top-notch projects focusing on the positive effects of conversation-based interventions on cognitive function (Otake-Matsuura et al., 2021; Sugimoto and Otake-Matsuura, 2022; Sugimoto et al., 2023), this project deepens the grasp of how language, memory, and cognition interact and offer valuable insights into cognitive functions on a broader scale. In the foreseeable future, harnessing language processing during extended conversations facilitated by robots could prove pivotal in attaining profound insights into the health of elderly individuals (Kumagai et al., 2022; Figueroa et al., 2023). This forward-thinking strategy represents a transformative stride toward optimizing healthcare for an aging population worldwide.

Expanding on research by Eyamu et al., portable EEG devices show the potential to identify nuanced cognitive abnormalities and brain alterations in MCI patients. Moreover, a recent study by Zheng et al. utilized EEG attributes like spectrum, complexity, and synchronization to aid AD diagnosis, emphasizing EEG's pivotal role in detecting neurological markers across conditions. These findings align with previous research and show that EEG data hold promise in extending early diagnosis and management of cognitive diseases, offering clinicians additional avenues to strengthen diagnostic precision and patient care (Al-Qazzaz et al., 2014; Maestú et al., 2019).

Addressing the challenging differentiation between Alzheimer's dementia and dementia with Lewy bodies (DLB), which typically involves invasive and resource-intensive techniques, Iannaccone et al. demonstrate an original study utilizing quantitative EEG (qEEG) for the early differential diagnosis. The study investigates the sensitivity and specificity of electroencephalography quantified using the statistical pattern recognition method (qEEG-SPR). The outlined technique significantly enriches the diagnostic landscape, offering a non-invasive and cost-effective approach. The findings underscore the efficacy of qEEG-SPR as a sensitive and specific tool for diagnosing dementia and distinguishing DLB from other forms of dementia in the initial stages. Crucially, this procedure holds promise as a tool that could be readily implemented in local care settings, addressing the practical challenges associated with current diagnostic methods and paving the way for improved identification and intervention strategies.

Next, Gil-Peinado et al. offer a comprehensive examination of factors associated with cognitive impairment. The research acknowledges the dynamic nature of mental health factors by integrating an up-to-date analysis standardizing the assessment of psychosocial, clinical, and lifestyle variables. This multifaceted approach advances readers' understanding of cognitive decline and lays a foundation for targeted dementia prevention strategies.

Finally, machine learning, particularly long-short-term memory (LSTM) algorithms, holds considerable promise for analyzing data from older adults and forecasting dementia trajectory. In the work by Huang, Huanget al., LSTM algorithms have successfully predicted the risk of MCI using longitudinal datasets. This screening method not only enables early intervention to delay the progression from MCI to dementia but also holds the potential to reduce the incidence and treatment costs (associated with dementia) in the long term.

In addition to the original works, the narrative of this Research Topic is further complemented by three insightful reviews. Liu et al. evaluate ultra-brief screening tools, enhancing delirium detection rates, particularly in older patients, potentially reducing adverse prognoses. The review searches databases such as the Cochrane Library, PubMed, and EMBASE, employing rigorous assessment tools like the COSMIN checklist and the QUADAS-2 tool to evaluate the diagnostic accuracy of ultra-brief screening tools for delirium. Significantly, the review identifies two instruments, 4 ‘A's test and UB-2, showcasing exceptional sensitivity in delirium screening. These results offer crucial insights for the early identification of delirium, offering significant relevance within clinical practice while extending scientific guidance to healthcare professionals.

Recognizing the vast challenges of early detection, the review by Wolf et al. discusses the limitations of traditional pen-and-paper tests and explores technological advancements in cognitive scoring methodologies. The review protocol adhered to the Preferred Reporting Items for Systematic Reviews and Meta-Analyses (PRISMA) guidelines, systematically searching electronic databases for peer-reviewed articles, examining visual processing among the MCI population, and reporting gaze parameters as potential biomarkers. While consistent with current trends in remote healthcare technology, the authors also examined studies that used non-commercial eye-tracking hardware to detect information processing problems in elderly people with MCI. In short, this high-quality literature synthesis suggests that eye-tracking-based paradigms can ameliorate screening limitations inherent in traditional cognitive assessments, paving the way for early AD detection.

The final review focuses on chromatic pupillometry, providing a unique viewpoint on particular photoreceptor functions in neurodegenerative diseases. Romagnoli et al. describe the use of chromatic pupillometry as a non-invasive method for assessing melanopsin retinal ganglion cells (mRGCs) in a variety of clinical contexts, including Parkinson's disease, rapid eye movement (REM) sleep behavior disorder, and Alzheimer's disease. The authors suggest that assessing mRGC-system functioning using chromatic pupillometry might serve as an early indicator of malfunction in neurodegenerative conditions characterized by circadian and sleep disturbance, setting the framework for future longitudinal cohort investigations.

In summary, the in-depth exploration of the Research Topic represents a significant step in understanding the subtle details of neurophysiological tools for identifying early signs of cognitive decline, Alzheimer's disease, and related dementias. The collective research articles set a path toward a future where integrating early detection, targeted intervention, and preventive strategies becomes fundamental in longevity research. Such direction holds the potential to revolutionize the approach to cognitive wellbeing, introducing an era where proactive and far-seeing neurophysiological measures redefine the landscape of healthcare and research (Moqri et al., 2023).

## Author contributions

AW: Conceptualization, Investigation, Project administration, Supervision, Writing—original draft, Writing—review & editing. KR: Writing—review & editing. ES-G: Writing—review & editing.
